# Enhanced Reactivity and Compound Mechanism of Mg/B Composite Powders Prepared by Cryomilling

**DOI:** 10.3390/ma15134618

**Published:** 2022-06-30

**Authors:** Chi Zhang, Qin Wang, Liying Tang, Fei Chen

**Affiliations:** State Key Laboratory of Advanced Technology for Materials Synthesis and Processing, Wuhan University of Technology, Wuhan 430070, China; zcontop@whut.edu.cn (C.Z.); wangqin0403@whut.edu.cn (Q.W.); zrwanda@163.com (L.T.)

**Keywords:** Mg/B composite powder, cryomilling, compound mechanism, reactivity

## Abstract

Boron is one of the highest energy density materials. The heat of boron is difficult to carry out due to its poor combustion performance. Magnesium (10 wt.%), acting as combustion adjuvant, is added into boron powder to improve the combustion performance. In this study, two kinds of boron powder were used as raw material, boron powder with an average size of 40 μm is named B1, and B2 has an average powder size of 3 μm. Mg/B composite powder was prepared though a cryomilling method. Two compound mechanisms for Mg/B composite powder were applied. For Mg/B1 composite powder, an Mg-coating structure on the surface of B was generated. For Mg/B2, a structure that small particles were agglomerated with Mg in the interior or on the surface of B was generated. Compared with either B powder or blended Mg/B powder, the reactivity of Mg/B composite powder by cryomilling is enhanced. In addition, the atomic ratio of Mg to B and activity content of Mg on the surface of Mg/B composite powder have great impacts on the improvement of reactivity.

## 1. Introduction

Aerospace vehicles typically use scramjets or ramjets as propulsion systems to obtain longer ranges at higher speeds. The potential performance of scramjets or ramjets for high-speed vehicles is currently explored and studied [1,2]. Boron-based fuel propellants are the highest energy solid propellants available and are promising to be the best energy source for scramjet engines [3]. The gravimetric heating value of B (58.5 MJ/kg) is almost three times that of hydrocarbon fuels and two times that of aluminum, which has prompted the interest of researchers in this field [4,5]. However, its poor combustion performance limits the application of B in industrial use. The existence of B_2_O_3_ on the surface of B impedes combustion performance. The melting temperature of B_2_O_3_ is 723 K, while the boiling temperature is 2316 K [6,7]. The high viscosity liquid B_2_O_3_ layer on the surface of B can prevent further combustion since B has no direct contact with oxygen [8,9]. Previous work has attempted to alleviate these challenges by combining boron with other metals [10], such as fluorinated compounds [11,12], carbides [13], and oxides [14,15,16]. These additives were added by milling and coating. Although shorter burn times and reduced ignition delays were reported, more than 10% of the additives were used in each case, reducing the overall energy density of the reacting material.

The addition of magnesium makes the ignition and combustion mechanism of the fuel more complex, which has attracted more attention in recent years. In Liu’s study [17], the ignition delay time of mixed Mg-B particles first decreased and then increased with increasing magnesium content. Liu [18] studied the combustion characteristics of composite Mg-B particles demonstrated that both magnesium content and ambient pressure impacts on the combustion performance. In addition, the study of magnesium diboride (MgB2) showed a two-stage combustion with a stable combustion showed yellow flame [19].

In this article, the cryomilling method was used to prepare the Mg and B (named as Mg/B) composite powder. Cryomilling can produce smaller particles and provide a cooling atmosphere during the process. It attains a relatively high specific surface area compared with the conventional methods. Ideal reactivity performance can be achieved through the cryomilling method. Compared with room temperature milling, cryomilling has significant advantages such as the following: First, small particles can be produced which powder agglomeration and welding to the milling media are avoided. Then, the oxidation of powder is significantly reduced since the milling process is performed in a nitrogen or argon atmosphere as B oxidation is the normally seen in the combustion of B. In this study, the combustion behavior is investigated by characterizing the oxidation of B. 

## 2. Experimental Procedure

**Figure 1:**
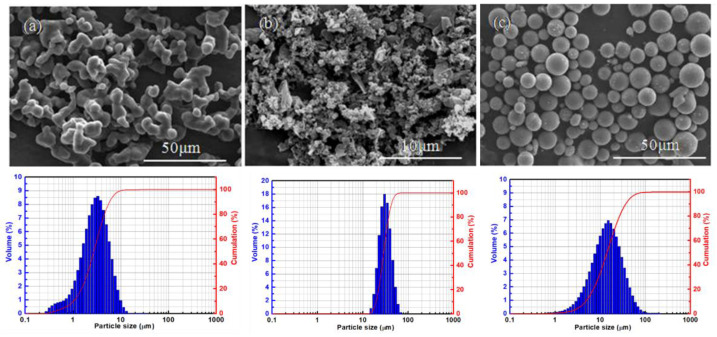
FESEM images of (**a**) B1, (**b**) B2, and (**c**) Mg raw powders.

As shown in Figure 1, two types of boron powder were used in this study: commercially available B powder (purity 99.9%) with an average size of 40 μm (named as B1) from ZhongPu Technology LLC (Beijing, China) and B powder (purity 99.9%) with an average size of 3 μm (named as B2) from Beijing General Research Institute of Mining and Metallurgy, Beijing, China. Another starting powder is commercially available Mg powders (purity 99.9%) with an average size of 15 μm. This was purchased from Shenzheng Tianyuan Surface Tech LLC, Shenzheng, China. A Malvern 2000 Mastersizer was used to analyze particle size distributions of raw powders.

The 01-HD vertical ball mill was used. B powder was weight-blended with 10 wt.% Mg powder. The blended powder was cryomilled in liquid nitrogen. Liquid nitrogen was continuously introduced into the equipment to maintain a relatively constant liquid level. The flowrate and pressure for the equipment was 35 mL/min and 0.05 Mpa, respectively. The ball-to-powder ratio (BPR) was 64:1 (*w*/*w*) and an impeller rotation speed was 500 rpm. The cryomilling time was 3 h, 6 h, and 9 h. The cryomilled powders were always handled in a vacuum drying oven for 4 h to reduce the chance of reaction with oxygen. The vacuum drying oven is set at 60 °C and −0.1 MPa. The specific surface areas of the prepared Mg/B composite powders are carried out on an Autosorb iQ (iQ-MP/XR) analyzer by Quantachrome Instruments. Samples were degassed at 200 °C under vacuum for 12 h with the powder mass ranged from 150 to 250 mg. In addition, the specific surface areas of the 9 h cryomilling Mg/B1 and Mg/B2 composite powders are 4.08 m^2^/g and 5.36 m^2^/g, respectively.

The phase compositions were identified by X-ray diffraction (XRD). XRD analysis was performed at an accelerating voltage of 40 kV and current of 40 mA; all scans were completed over the range 10~90° in 1 h. The microstructure was investigated in detail using field emission scanning electron microscopy (FESEM; FEI Quanta FEG250 FEI company, Hillsboro, OR, USA); it was carried out at an accelerating voltage ranging from 0.5 to 30 kV, the samples were kept at a pressure of 10^−8^ Torr. The content of Mg and Fe was tested by plasma emission spectroscopy (ICP; Optima 4300DV from Perkin Elmer, Shelton, CT, USA), where oxygen and nitrogen were detected by TC 600; the ICP test performed at a wavelength ranging from 165 to 1151 nm with 30 Psi Gas flow rate. The microstructure was also observed through transmitted electron microscope (STEM; JEM-2100F from JEOL, Toykyo, Japan) at a 200 kV accelerating voltage. The image analyzer system (DS-5M from Puye LLC., Shanghai, China) was operated at a flow rate ranging from 3000 to 8000 mL/min. X-ray photoelectron spectroscopy (XPS; VG Multilab 2000 from Bruker, Billerica, MA, USA) was recorded at room temperature with Al Kα source (1486.6 eV) and step size 0.05 eV. The thermal performances of samples were investigated by a simultaneous thermal analyzer (A449c/3/G). The heating temperature ranged from room temperature to 1000 °C in the flowing stream of oxygen while the heating rate was 20 °C/min.

## 3. Results and Discussion

### 3.1. Synthesis and Characterization of Mg/B Composite Powders

**Figure 2:**
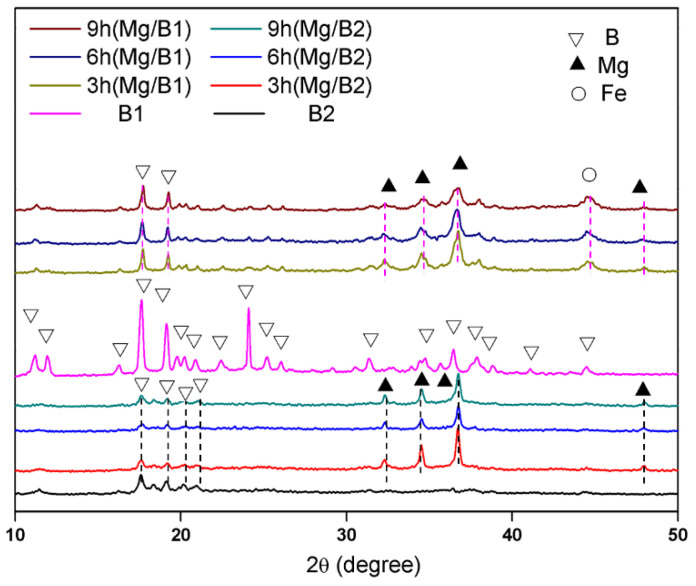
XRD patterns illustrating Mg/B composite powders.

Figure 2 shows the XRD patterns of B and cryomilled Mg/B composite powders. The phases of cryomilled powder are metallic Mg (PDF#65-3365), B (PDF#31-020), and metallic Fe (PDF#06-0696). No compounds of Mg and B were detected. With the increase in cryomilling time, the peak value of Mg decreases. Moreover, the peak value of B is much lower compared with the original B powder, which cryomilling breaks the order of to a certain degree. There is a vital difference between the two kinds of Mg/B composite powders. The intensity of the diffraction peak of Mg in Mg/B1 composite powder is much weaker and broader. It can be inferred that the crystallinity of Mg in Mg/B1 composite powder is lower compared with the original powder, the crystallinity of Mg and B in two Mg/B composite powders prepared by cryomilling are reduced. Moreover, the crystallinity of B in Mg/B1 is significantly higher compared with Mg/B2 composite powder. 

Mg/B composite powder is composed of five elements, B, O, N, Mg, and Fe, since the volume of others micro-constituents is low enough to ignore. The analysis results for O, N, Mg, and Fe content are summarized in Table 1 and Table 2. The content of B was calculated by subtracting the content of the other four elements due to its measuring difficulty. 

Powder contamination caused by the processing media or the atmosphere is an inherent characteristic of mechanical milling. N is introduced into the powder during the milling for the following reasons: first, liquid nitrogen is the milling media during cryomilling; in addition, powder has a high tendency to react with O [20]. According to Lavernia’s study, the results showed an increase in specific surface area by 10 times after cryomilling [21]. The increasing surface area improves the chemical activity of powders and facilitates the adsorption of gaseous atoms. The amount of N (below 2 wt.%) increases with cryomilling time increases. The content of N in Mg/B1 composite powder is relatively high. The amount of O is about 3 wt.%. The slight increase in O is corresponding to the possible opportunity to ingress air in the process. According to several studies of cryomilling, the content of N can be reduced to 0.024 wt.% after the degassing process or it can be collected in an argon glove box where O reached about 0.5 wt.% [22,23]. As Table 1 and Table 2 show, the amount of Fe increases with the increases in cryomilling time. The Fe contamination is caused by the wear of the stainless steel milling balls and shaft during cryomilling. Thus, the cryomilling time should be controlled. In this study, cryomilling should be kept for 9 h since the content of Fe increases to a substantial amount beyond this point. In addition, Fe is an energy material that can be used as an addition to the propellant [23]. The content of Mg is less than 10 wt.%, a reduction as a result of introducing impurities.

**Figure 3:**
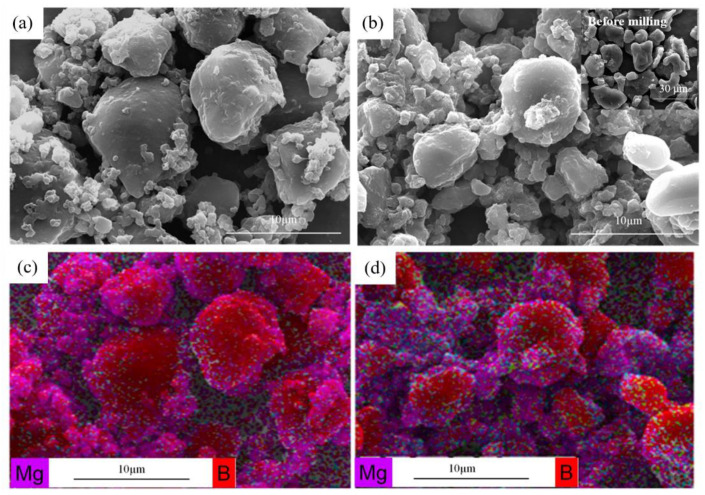
FESEM images of Mg/B1 composite powders cryomilling for (**a**) 6 h, (**b**) 9 h, and corresponding EDS images (**c**,**d**).

**Figure 4:**
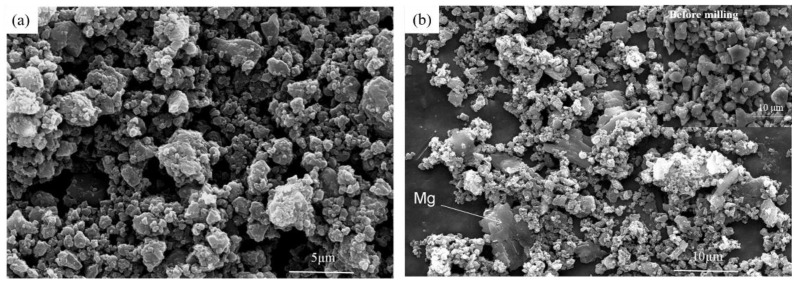
FESEM images of Mg/B2 composite powders cryomilling for (**a**) 3 h and (**b**) 9 h.

The morphology of two Mg/B composite powders is irregular and rugged as shown in Figure 3 and Figure 4. As the cryomilling time increases, particle size tends to be smaller with Mg tending to be more homogeneous, spreading on the surface of B particles. It can be observed that big powder particles were coated by small particles in Mg/B composite powder. However, for Mg/B2 composite powder, there is a vital number of small particles that are not agglomeration (shown in Figure 4b). Compared with Mg/B1 composite powder, the particle size of Mg/B2 composite powder is smaller.

**Figure 5:**
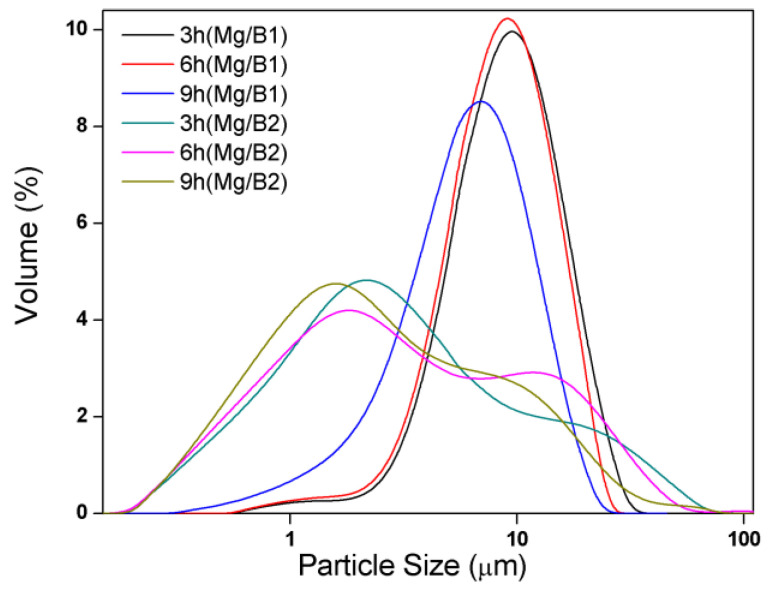
Particle size distribution of Mg/B composite powders.

Particle size has a great effect on the combustion behavior of B particles. The particle size of Mg/B1 composite powder after cryomilling for 3 h, 6 h, and 9 h are 3.944 μm, 3.233 μm, and 3.125 μm, respectively. Compared with the raw materials, the particle size decreased. Compared with the raw materials, the particle size of Mg/B2 composite powder decreased over 80%. The particle size of Mg/B2 composite powder after cryomilling for 3 h, 6 h, and 9 h are 2.651 μm, 2.537 μm, and 2.111 μm, respectively. It only decreases with a slight degree compared with B2. The cryomilling can be divide into three steps: (1) The microcracks expand under the repeated impact of the grinding balls until they penetrate the entire particle, the particles are broken and refined. (2) The role of fracture becomes weaker, as shown by the rate of particle size reduction becomes slower with large particles are generated. (3) Fracture and cold welding reach a dynamic equilibrium, the particle size of composite powder fluctuates around a certain value, the particle refinement is no longer obvious. It is difficult to generate smaller particles under this circumstance [24]. In Figure 5, Mg/B2 composite powder tends to be agglomerated since multiple peaks can be observed. This is in accordance with the SEM analysis.

**Figure 6:**
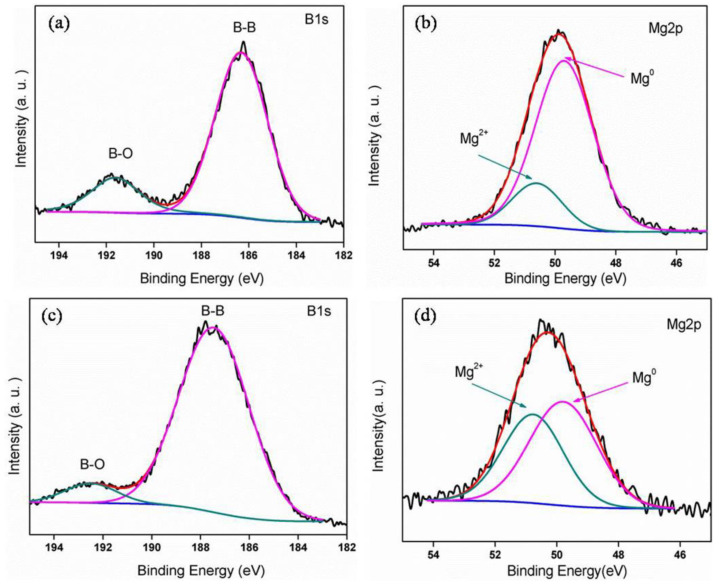
XPS spectrum of (**a**) B1s, (**b**) Mg2p of Mg/B1, (**c**) B1s, and (**d**) Mg2p of Mg/B2cryomilled for 9 h.

The element Mg and B in two Mg/B composite powders were detected by XPS (shown in Figure 6). Characteristic peaks of B_2_O_3_ and B can be identified by two fitting peaks at 192.3 eV and 186.4 eV of the B1s peak. The Mg2p is composed of two peaks, where the 50.4 eV peak corresponds to Mg and the 49.4 eV peak corresponds to Mg^2+^ in MgO [25]. The activity content of B and Mg are defined as the ratio between zero oxidation area to the whole area. The activity content of B in Mg/B1 composite powder (around 85%) is much lower than Mg/B2 composite powder (above 93%). On the contrary, the activity content of Mg in Mg/B1 composite powder (approach 90%) is much higher than Mg/B2 composite powder (about 60%). The activity content of B and Mg are summarized in Table 3.

### 3.2. Microstructure and Compound Mechanism of Mg/B Composite Powders

**Figure 7:**
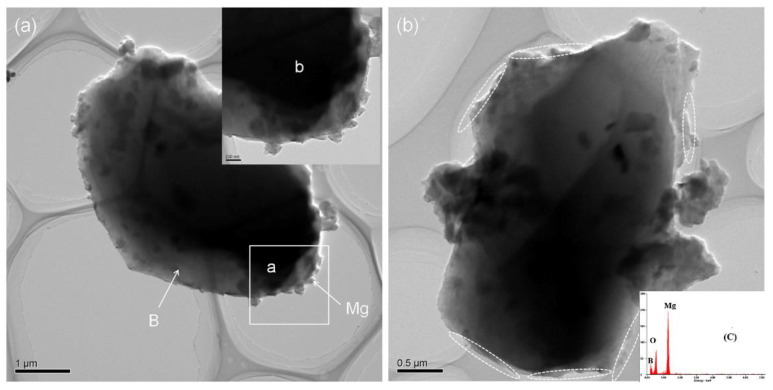
TEM images of (**a**) Mg/B1 cryomilled for 6 h, (**b**) and Mg/B1 cryomilled for 9 h.

In Figure 7a, picture b is the enlarged view of part a marked with a white box. It indicates that in the process of cryomilling, brittle fracture occurs due to smaller B particles (marked as B in Figure 7a) where Mg particles have a tendency to be embedded in the hole and pits of B particles [25]. With the increase in cryomilling time, Mg tends to diffuse to the surface of B particles as shown in Figure 7b. In Figure 7c, the EDS results from the surface area of B particles show a obvious Mg peak. It verify the Mg diffusion phenomenon on surface of B particles.

**Figure 8:**
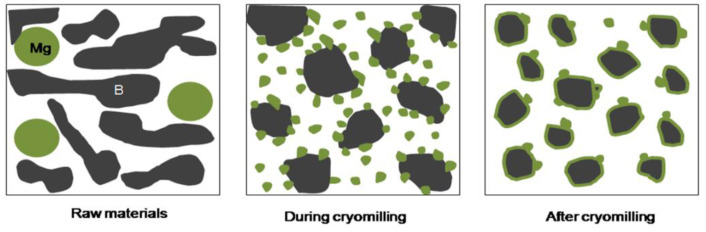
Illustration of the compound mechanism of Mg/B1 powders.

During cryomilling process, the compound mechanism of Mg/B1 powder was divided into several continuous stages as shown in Figure 8. First, B particles and Mg particles (marked as B and Mg in Figure 8) are severely deformed, B particles tend to become smaller with holes and pits on the surface while Mg particles present a small smooth flake morphology. Then, Mg particles are crafted into smaller particles due to the shear strength between Mg particles and B particles or Mg and milling balls [26]. When the small Mg particles meet the hole and pits of B particles, it is easy for Mg particles embedded in B particles. With the shear and strike impact during the milling process, Mg particles are uniformly distributed on the surface of B particles.

**Figure 9:**
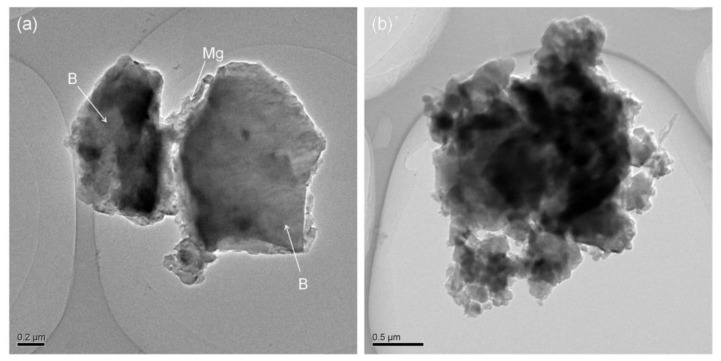
TEM images of (**a**) Mg/B2 cryomilled for 6 h, (**b**) and Mg/B2 cryomilled for 9 h.

For Mg/B2 composite powder, it is quite different due to the particle size of starting B powders. Since most particles of B2 (marked as B in Figure 9a) are relatively small after cryomilling, B particles tend to be aggregated as shown in Figure 9a. In Figure 9b, it is noteworthy that the agglomeration of powder is more obvious with the increase in cryomilling time. However, With the increase in cryomilling time, Mg particles on the surface of B particles can be transferred into the interior of Mg/B2 composite powders due to powder agglomeration.

**Figure 10:**
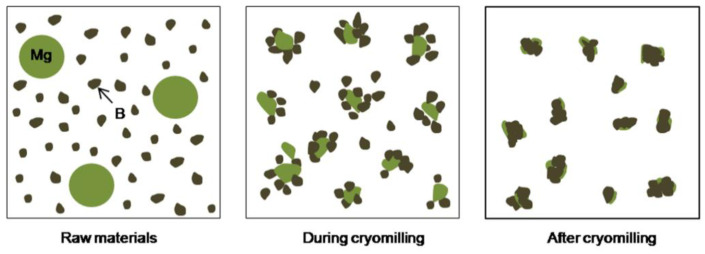
Illustration of the compound mechanism of Mg/B2 powders.

For Mg/B2 powders, a different process occurred as shown in Figure 10. Since particle size of B2 is much smaller than Mg particles (marked as B and Mg in Figure 10) Mg particles present good ductility. Smaller B particles tend to aggregate with the bind of Mg particles after cryomilling. In addition, some Mg particles are embedded into the hole and pits of B particles. With the increase in cryomilling time, the particle size does not change obviously, while Mg particles on the surface or inside the composite powder have a tendency of tiling [26].

### 3.3. Enhanced Reactivity of Mg/B Composite Powders

**Figure 11:**
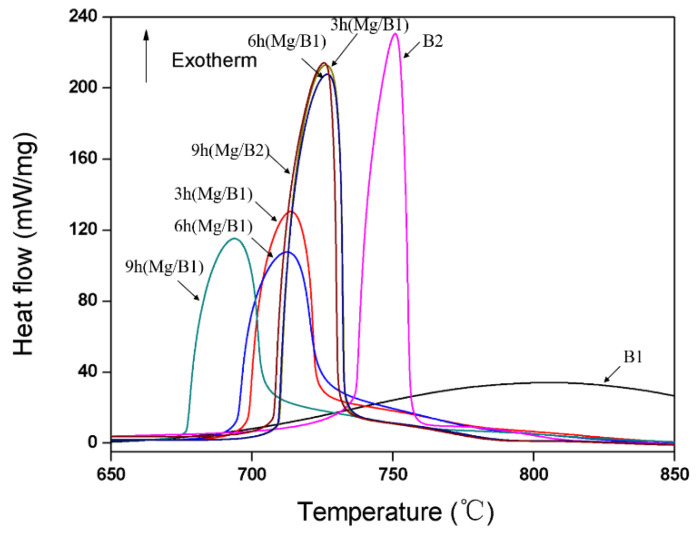
DSC curves of raw B powders and Mg/B composite powders.

Figure 11 shows the TG/DSC data of Mg/B composite powders compared with the raw B powders. There is a big exothermic peak between 700 °C and 800 °C. The exothermic peak for Mg/B1 composite powder presents a higher and sharper morphology compared with the exothermic peak of B1 powder. The peak temperature of the main peak for Mg/B1 composite powder after 3 h, 6 h, and 9 h of cryomilling are 713.7 °C, 712.6 °C, and 693.8 °C, respectively. Compared with the B1, whose main peak is 806.5 °C, the exothermic peak of Mg/B1 composite powder appears at a lower temperature. The temperature of the exothermic peak decreases with an increase in cryomilling time. Compared with B2 powder, the exothermic peak for Mg/B2 composite powder demonstrates a shorter and broader morphology due to the addition of Mg and a wide range of particle size after cryomilling. The peak temperature of the main peak changes from 750 °C to 726 °C. It is believed that the reactivity of B particles in Mg/B composite powder is enhanced from cryomilling. For Mg/B2 composite powder, the temperature of the exothermic peak has no significant changes with the increases in cryomilling time.

As shown in Table 4, the released heat for Mg/B1 composite powder after cryomilling is slightly decreased. This is inevitable with the introduction of impurities such as elements N, O, and Fe. For Mg/B2 composite powder, the released heat becomes higher after cryomilling since the reactivity of B particles is enhanced. In related studies [15,16], the numerical value of released heat is relatively higher. It can be concluded that the method used in this study has the potential to improve the oxidation of B particles.

It can be inferred that the oxidation peak temperature of B is positively correlated with the ignition temperature of B. Therefore, the combustion behavior of Mg/B composite powder is improved by cryomilling. From the results in Table 4, it can be concluded that the combustion behavior of Mg/B1 composite powder is better than Mg/B2 composite powder. In addition, combustion behaviors from other research are summarized in Table 5; it is obvious that the Mg/B composite powder prepared in this study has better combustion behavior.

The crystallinity and particle size are key factors in the oxidation behavior of B particles [30]. Considering crystallinity (shown in Figure 2) and particle size (shown in Figure 5), the oxidation temperature of B particles in Mg/B2 composite powder is expected to be lower than Mg/B1 composite powder.

Taking the different compounding mechanisms into consideration, the surface of Mg/B composite powder impacts the combustion behavior. The form and content of Mg particles and B particles on the surface of Mg/B composite powder were characterized by XPS. The atomic ratio of Mg particles to B particles on the surface is listed in Table 6. Compared with the blend ratio (0.050), the ratio of cryomilling Mg/B composite powder is much higher. It is possible that Mg particles are accumulated on the surface of the composite powder, which leads to an Mg-coating structure of B particles. Moreover, the atomic ratio of Mg particles to B particles in Mg/B1 and Mg/B2 composite powders increase with the increase in cryomilling time. However, the atomic ratio of Mg particles to B particles in Mg/B1 composite powder is much higher. The results are consistent with the compounding mechanisms of two Mg/B composite powders. For Mg/B2 composite powder, part of Mg particles on the surface of B particles is transferred into the interior of Mg/B2 composite powder where part of the B particles tends to agglomeration.

The reason Mg can improve the combustion behavior of B attribute to the heat released by the reaction is as follows: 2Mg + O_2_ = 2MgO, which can preheat B particles. In addition, the reaction: 3Mg + B_2_O_3_ = 2B + 3MgO can remove the oxide layer of B particles. As shown in Table 3, the activity content of Mg particles in Mg/B1 composite powder is much higher than Mg/B2 composite powder, while the activity content of B particles is on the contrary. At the cost of reduction in the activity content of Mg, Mg/B2 composite powder possess high activity content of B with the following reaction: 3Mg + B_2_O_3_ = 2B + 3MgO.

**Figure 12:**
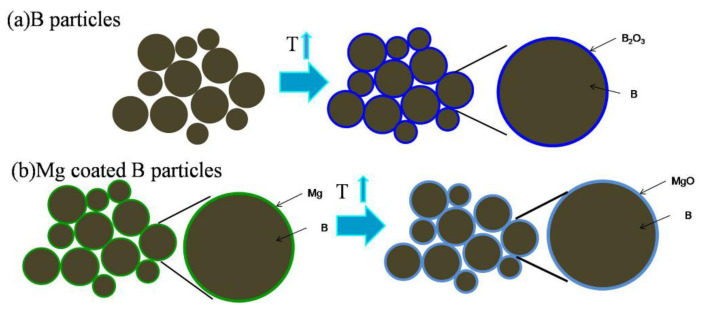
Models of B and Mg/B powder oxidation.

In this study, simply adding Mg into B makes the oxidation temperature of B higher, where 806.5 °C is increased to 834.0 °C for B1, and 750.9 °C is increased to 767.3 °C for B2. This indicates that adding Mg into B cannot improve the reactivity of B. In addition, the study of B shows that the combustion performance of B with a coating structure is improved because heat released by the reaction between Mg and O heats up B particles. In the preparation of Mg/B composite powder, the atomic ratio of Mg to B and activity content of Mg on the surface have great effects on the reactivity of oxidation. As shown in Figure 12, Mg/B composite powder generates a layer of MgO, instead of the oxide layer of B, which tends to become liquid and has a high viscosity. The phenomena make the B particles aggregate, resulting in inhibition of further B oxidation.

## 4. Conclusions

In this study, two different compound mechanisms were applied to obtain two types of Mg/B composite powders. The influence of magnesium addition on the composite process, heat release, and the exothermic peak temperature for ignition performance were obtained. The compound mechanisms for Mg/B composites were revealed based on the detail experiment. The main conclusions are summarized below:

(1) Impurities Fe, N, and O were introduced into Mg/B composite powders via cryomilling. No obvious impacts on energy density since Fe is an energy material. The study of cryomilling shows that O and N can be removed after the degassing. The Fe content increases with the increase in cryomilling time, consequently cryomilling time should be limited;
(2) By adding magnesium and cryomilling, the reaction temperature between B and O is improved by more than 90 °C for Mg/B composite powder(particle size 3.125 μm) and 24 °C for Mg/B composite powder(particle size 2.111 μm). The exothermic peak for Mg/B composite powder(particle size 3.125 μm) composite powder is higher and sharper than its starting B powder. The reaction temperature decreases with the decrease in particle size.
(3) The combustion behavior of Mg/B composite powder is improved by cryomilling. In particular, the 9 h cryomilling Mg/B composite powder(particle size 3.125 μm) composite powder shows an extremely good combustion behavior with a 13,237 J/g release heat and relatively low exothermic peak temperature at 693.8 °C;
(4) Comparing the physical properties of Mg/B composite powder(particle size 3.125 μm) and Mg/B composite powder(particle size 2.111 μm), the results indicate that the activity content of Mg on the surface plays a dominant role in the improvement of reactivity.

## Figures and Tables

**Table 1:** The content of Mg/B1 composite powders cryomilled for 3 h, 6 h, and 9 h.

Content [wt.%]	3 h	6 h	9 h
Mg	9.32	9.26	9.63
Fe	0.21	0.30	0.42
O	3.01	3.04	3.29
N	1.80	1.93	1.97
B	85.66	85.47	84.69

**Table 2:** The content of Mg/B2 composite powders cryomilled for 3 h, 6 h, and 9 h.

Content [wt.%]	3 h	6 h	9 h
Mg	9.87	9.56	9.46
Fe	0.21	0.21	0.32
O	2.37	2.48	2.66
N	1.37	1.54	1.63
B	86.08	86.01	85.77

**Table 3:** The activity content (%) of Mg and B in Mg/B composite powders.

Cryomilling Time (h)	3	6	9
B (Mg/B1)	86.08	85.41	84.37
Mg (Mg/B1)	88.38	87.65	89.59
B (Mg/B2)	92.90	93.07	92.86
Mg (Mg/B2)	60.09	54.38	55.87

**Table 4:** The exothermic peak and released heat.

Sample	Temperature (°C)	Heat Release (J/g)
B1	806.5	15,325
Mg/B1 cryomilled for 3 h	713.7	12,830
Mg/B1 cryomilled for 6 h	712.6	13,555
Mg/B1 cryomilled for 9 h	693.8	13,237
B2	750	10,079
Mg/B2 cryomilled for 3 h	726.0	12,601
Mg/B2 cryomilled for 6 h	726.7	11,305
Mg/B2 cryomilled for 9 h	726.2	11,198

**Table 5:** The combustion behavior between B-based materials prepared by different method [27,28,29].

Method	Materials	Temperature (°C)	Heat Release (J/g)
Cryomilling	Mg/B	693.8	13,237
Sintering	MgB_2_/B	810	15,600
Milling	MgB_2_/B	750	8500
Ultrasonic dispersion	HMX/B	607	9110
Laser ignition	Mg-Al/B	749	7993

HMX: cyclotetramethylenetetranitramine.

**Table 6:** The atomic ratio of Mg to B on the surface of Mg/B composite powders.

Cryomilling Time (h)	0	3	6	9
Mg/B1 composite powders	0.05	0.69	0.83	1.12
Mg/B2 composite powders	0.05	0.09	0.15	0.17

## Data Availability

Data available on request due to restrictions eg privacy or ethical.
